# Biorecognition-Based Nanodiagnostics: Maltotriose-Functionalized Magnetic Nanoparticles for Targeted Magnetic Resonance Imaging of Bacterial Infections

**DOI:** 10.3390/bioengineering12030296

**Published:** 2025-03-15

**Authors:** Junshan Wan, Chuqiang Yin, Xiaotong Chen, Keying Wu, Chonghui Zhang, Yu Zhou, Yugong Feng, Jing Chang, Ting Wang

**Affiliations:** 1 Department of Clinical Medicine, Qingdao Medical College, Qingdao University, Qingdao 266071, China; wanjunshan@qdu.edu.cn (J.W.); chuqiangyin@qdu.edu.cn (C.Y.); chenxiaotong2@qdu.edu.cn (X.C.); doctorzch@qdu.edu.cn (C.Z.); yuzhou@uor.edu.cn (Y.Z.); fengyugong@qdu.edu.cn (Y.F.); 2 College of Marine Life Science, Ocean University of China, Qingdao 266003, China; 21230613080@stu.ouc.edu.cn; 3 Laboratory for Marine Drugs and Bioproducts, Qingdao Marine Science and Technology Center, Qingdao 266003, China; 4 Department of Health and Life Science, University of Health and Rehabilitation Sciences, Qingdao 266113, China

**Keywords:** molecular imaging, magnetic resonance imaging, magnetic nanoparticles, bacterial infection

## Abstract

Bacterial infections remain a global healthcare challenge, requiring precise diagnostic modalities to guide therapeutic interventions. Current molecular imaging agents predominantly detect nonspecific hemodynamic alterations and lack pathogen-specific targeting capabilities for magnetic resonance imaging (MRI). Leveraging the selective bacterial uptake of maltotriose via the maltodextrin transport pathway, we engineered maltotriose-functionalized magnetic nanoparticles (Malt-MNPs) as a novel MRI contrast agent. Basic physicochemical characterization confirmed the nanosystem’s colloidal stability, biocompatibility, and superparamagnetism (saturation magnetization > 50 emu/g). In a rat bacterial infection model, intravenously administered Malt-MNPs selectively accumulated at infection sites, inducing a >50% MRI signal change within 24 h while exhibiting minimal off-target retention in sterile inflammatory lesions (<10% signal change). This specificity enabled clear MRI-based differentiation between bacterial infections and noninfectious inflammation. These findings provide a promising strategy for clinical translation in infection imaging and treatment.

## 1. Introduction

Infectious diseases are one of the major global threats to human health. Approximately 7.7 million people died from infectious diseases in 2019, accounting for over 13% of global deaths [1,2]. Gram-positive bacteria (e.g., *Staphylococcus aureus* (*S. aureus*)) and Gram-negative bacteria (e.g., *Escherichia coli* (*E. coli*)) often cause pneumonia, sepsis, urinary tract infections, and postoperative infections [3,4,5]. Further, the increasing severity of antibiotic resistance has complicated the situation worldwide [6,7,8]. Recently, the incidence of hospital-acquired infections has abruptly risen with the widespread implementation of minimally invasive surgery, organ transplantation, long-term hospitalization, and catheter-based medical procedures [3,9]. Although these procedures have improved treatment outcomes, they have also increased the risk of infection, making hospitals the main sites for the transmission of drug-resistant pathogens [10]. According to the World Health Organization, diagnosis must be a fundamental component of any effective public health system, and the development of rapid, reliable, and affordable testing is crucial for developing treatment methods for infectious diseases [11].

Currently, the diagnosis of infectious diseases relies on traditional microbiological methods and imaging tests. Microbiological methods such as microscopy, microbial culture, and molecular biology techniques (e.g., polymerase chain reaction and mass spectrometry) are essential for the definitive diagnosis of infections. However, they still have considerable limitations in practice [12,13,14]. While microscopic examination offers limited pathogen detection accuracy, microbial culture is time-consuming, which can delay the treatment of patients with acute or severe infections [15]. Moreover, molecular biology techniques such as polymerase chain reaction and mass spectrometry require advanced equipment although they offer high sensitivity and specificity, and they often provide the overall pathological status of the patient, making it difficult to accurately assess local pathological changes at the infection site, especially in deep tissue or early-stage infections [16,17,18]. More importantly, traditional microbiological methods can lead to false-negative results for pathogens that are difficult to culture or are present at low concentrations, limiting their effectiveness in clinical diagnosis [11,13].

Imaging technologies play a crucial role in the diagnosis of infectious diseases, particularly in assessing deep infections. However, traditional X-rays and computed tomography (CT) scans rely on tissue structural changes caused by infection, such as fluid accumulation and inflammatory necrosis; their sensitivity is insufficient for early-stage infections or lesions with no discernable structural changes [19,20]. Owing to the rapid development of molecular imaging technologies, imaging agents already applied in positron emission tomography (PET) and single-photon emission computed tomography (SPET) (e.g., 18F-fluorodeoxyglucose) can offer higher sensitivity compared to that achieved using structural imaging tools [21,22,23]. Nevertheless, the aforementioned imaging techniques share an inherent limitation: they involve a degree of radiation exposure, restricting their applicability in clinical scenarios that require repeated evaluations [24,25]. In contrast, magnetic resonance imaging (MRI) has attracted considerable interest as a nonionizing imaging technique, owing to its high resolution in soft tissue imaging and minimal side effects. Via the use of functionalized contrast agents, MRI not only provides detailed anatomical information but also deeply reflects local pathophysiological features, enabling the early diagnosis and precise localization of diseases [26,27,28,29]. However, MRI has limitations in differentiating infectious lesions from sterile inflammation, especially in the absence of clear inflammatory responses or structural changes, limiting its diagnostic capability for early-stage infections [30].

Magnetic nanoparticles (MNPs) have become a prototypical transverse relaxation time (T2)-weighted MRI contrast agent, owing to their good biocompatibility [31,32,33,34], superior photothermal characteristics, and high metabolic rate [35,36]. Additionally, MNPs exhibit superparamagnetism, high magnetization, and no remanent magnetism or coercivity, ensuring that they respond strongly to the external magnetic field during MRI but do not retain magnetization after the magnetic field is removed, thereby minimizing aggregation and enhancing stability. They have been employed in various fields, including in vivo tissue imaging, real-time in vivo diagnostics, drug delivery, photothermal therapy, and tissue engineering [29,37,38,39]. However, MNPs without any functionalization often exhibit poor stability in vivo and tend to aggregate in biological environments, which can lead to dipole–dipole interactions between nanoparticles. These interactions can increase the superparamagnetic (SPM) blocking volume and alter the magnetic anisotropy, thereby limiting their imaging performance and reducing the signal contrast in MRI [40,41,42]. Additionally, they lack sufficient targeting capabilities, making it difficult to accurately identify and localize infection sites, further restricting their applications in precision medicine. Consequently, the functionalization of MNPs has become a key strategy to enhance their stability and impart targeting abilities for improving their imaging performance.

Carbohydrates serve as crucial energy sources for bacteria, which utilize both glucose and maltodextrins. Bacteria internalize maltodextrins via maltodextrin transporters and the phosphotransferase system [43,44,45]. However, the chain length and structure of maltodextrins remarkably impact their stability and uptake efficiency in vivo. Maltodextrins with different chain lengths and terminal modifications have been shown to exhibit varying stability in serum, which may influence their effectiveness as tracers [46]. For instance, probes derived from maltotriose (e.g., Cy7-1-maltotriose) show higher accumulation and stronger SI compared to those derived from maltohexose, particularly at infection sites in mouse models [47]. Therefore, as an oligosaccharide with excellent water solubility and biocompatibility, maltotriose has garnered immense interest in biomedicine. Unlike monosaccharides (e.g., glucose) and disaccharides (e.g., sucrose), maltotriose is minimally taken up by normal human cells, whereas most bacteria, especially Gram-positive bacteria (such as *S. aureus*) and Gram-negative bacteria (such as *E. coli*), can take up maltotriose through specific sugar transport proteins (e.g., maltodextrin transporters or phosphotransferase system) on their surface for metabolism and utilization [44]. Therefore, maltotriose can precisely target and accumulate at infection sites by binding to bacterial surface sugar receptors, notably enhancing imaging specificity and sensitivity. This specific uptake mechanism makes maltotriose an ideal targeting molecule, particularly in nanomaterial modifications to promote nanoparticle accumulation at lesion sites [47,48,49,50].

**Scheme 1:**
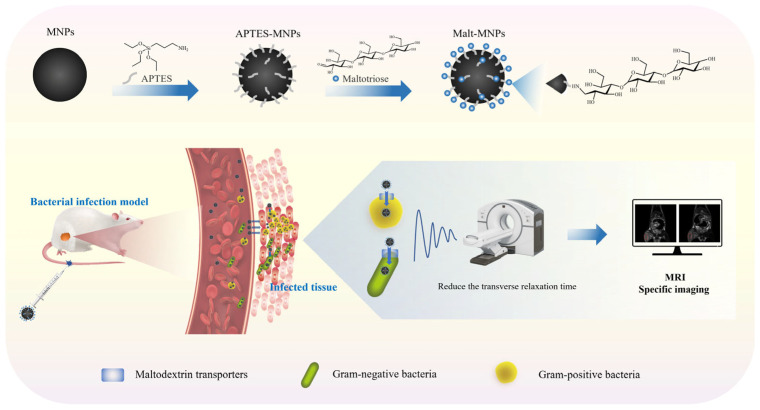
Design of nanoprobes for specific detection of bacterial infections via magnetic resonance imaging.

In this study, we developed a highly sensitive and specific diagnostic tool based on the targeting capability of maltotriose and the MRI performance of MNPs, namely maltotriose-coated MNPs (Malt-MNPs), to enable the early and precise imaging of infection sites. As depicted in Scheme 1, MNPs were functionalized with 3-aminopropyltriethoxysilane (APTES), resulting in APTES-MNPs with highly reactive amino groups. Subsequently, maltotriose was covalently attached to the surface of APTES-MNPs through a Schiff base reaction between the aldehyde groups of maltotriose and the amino groups of APTES-MNPs, producing Malt-MNPs. The size distribution, surface charge, magnetic properties, and chemical structure of the synthesized nanoparticles were comprehensively characterized. Additionally, the biocompatibility and targeting capability of Malt-MNPs were systematically evaluated. The results show that Malt-MNPs selectively bind to bacteria and remarkably reduce T2, enabling high-contrast MRI. In vivo experiments using deep muscle tissue infection and sterile inflammation models further demonstrate the ability of Malt-MNPs to differentiate between bacterial infection and sterile inflammation. Unlike traditional MRI contrast agents that rely on local vascular permeability, Malt-MNPs exhibit high signal contrast and specificity at bacterial infection sites, reducing nonspecific aggregation and systemic toxicity. Our study shows that this enhances the sensitivity and accuracy of MRI and expands the potential applications of molecular imaging technologies in the diagnosis and treatment of infectious diseases.

## 2. Materials and Methods

### 2.1. Synthesis of MNPs

MNPs were synthesized using a modified version of the classic chemical coprecipitation method [51]. Briefly, under N_2_ protection and vigorous mechanical stirring, 21.62 mg FeCl_3_·6H_2_O and 13.34 mg FeSO_4_·7H_2_O were dissolved in 20 mL deoxygenated water. Subsequently, the solution was heated to 60 °C, followed by the slow addition of 2.5 mL concentrated ammonia solution to obtain a pH value in the range of 9.0–10.0. The reaction was maintained for 30 min. Consequently, the temperature was increased to 80 °C, and the reaction was continued for another 30 min before cooling to room temperature. Throughout the reaction, the solution was kept under a nitrogen atmosphere. The black precipitate was washed with deionized water and ethanol using magnetic separation, followed by freeze-drying to obtain the product.

### 2.2. Synthesis of Malt-MNPs

First, MNPs were functionalized with APTES to introduce amino groups on the surface. Using the classic method [52], 100 mg MNP powder was dispersed in a mixture of 100 mL ethanol and 1 mL water, followed by the dropwise addition of 180 μL APTES. The solution was mechanically stirred for 7 h, centrifuged to collect the precipitate, which was washed three times with water and ethanol, and freeze-dried for later use. Under N_2_ protection, 15 mg APTES-MNP powder was dispersed in 3.75 mL deionized water, sonicated, and mixed with a solution of maltotriose (20 mg/mL, 5 mL) dissolved in deionized water. The mixture was stirred continuously at 70 °C for 6 h. NaBH_3_(CN) solution (4 mg/mL, 250 μL) was then added, and the reaction was conducted at room temperature for 12 h. The mixture was centrifuged (21,130× *g*, 20 min) and purified three times to obtain the maltotriose-modified MNP solution (Malt-MNPs), which was then freeze-dried.

### 2.3. Characterization of Malt-MNPs

The morphology and size of MNPs and Malt-MNPs were observed using TEM (JEM-2100 F, JEOL, Akishima City, Japan) at 100 kV. FTIR spectra were recorded using an iS50 FTIR spectrometer (Thermofisher, Waltham, MA, USA). Raman spectra were recorded using DXR2 Microscopical Laser Raman spectroscopy (Thermofisher, Waltham, MA, USA). DLS and zeta potential measurements were performed using an Omni multiangle particle size and high-sensitivity zeta potential analyzer (Brookhaven Inc., Nashua, NH, USA). The magnetic properties of the nanoparticles were measured at room temperature using a vibrating sample magnetometer (VSM-130, Changchun Yingpu Magnetoelectric Technology Development Co., Ltd., Changchun, China). The T2-weighted MRI of nanoparticle solutions at different concentrations (2 mL in ultrapure water) was obtained using a SIGNA Pioneer 3.0T MRI system (General Electric Company, Boston, MA, USA). The T2 values of different concentrations of nanoparticle solutions were measured using low-field nuclear magnetic resonance (MacroMR12-150H-I, Suzhou Niumag Analytical Instrument Corporation, Suzhou, China). The scanning parameters were as follows: pulse sequence: CPMG; operating frequency (SF): 12 MHz; sweep width (SW): 200 kHz; repetition time (TW): 2000 ms; echo time (TE): 0.100 ms; number of echoes (NECH): 4000; and data points collected (TD): 80012. The T2 values corresponding to series concentrations (mM) were fitted to obtain the r_2_ values (mM^−1^·s^−1^) of MNPs and Malt-MNPs.

### 2.4. Bacterial Culture

*S. aureus* (ATCC 25923) was purchased from the American Type Culture Collection (ATCC). All bacterial culture reagents (such as TSA agar plates) were obtained from Dalian Meilun Biotechnology Co., Ltd., Dalian, China. Bacterial suspension was spread onto TSA agar plates and incubated at 37 °C for 12 h. Consequently, single colonies were selected and cultured in a TSB liquid medium for 12 h (250 rpm, 37 °C) to reach the logarithmic phase. The bacterial suspension was washed twice and resuspended in PBS buffer for subsequent use. The bacterial concentration was determined by measuring the optical density at 600 nm.

### 2.5. In Vitro Imaging of Bacteria

The Malt-MNP solution (2.5 mM) was incubated with *S. aureus* suspension (10^7^ CFU) for 1 h. This mixture, along with an equal volume of PBS, MNPs, and *S. aureus* alone, was subjected to T2-weighted MRI using SIGNA Pioneer 3.0T (General Electric Company, Boston, MA, USA). The bacterial suspension (10^7^ CFU) mixed with the incubated solution was then centrifuged (2000 rpm, 5 min). The resulting pellet was fixed with glutaraldehyde, followed by ultrathin sectioning and TEM (JEM-2100 F, JEOL).

### 2.6. Infection Animal Models

Animal procedures were performed following the guidelines and regulations approved by the Institutional Animal Care and Use Committee. Male SD rats (220–250 g body weight) were obtained from Jinan Pengyue Experimental Animal Breeding Co., Ltd., Jinan, China. The rats were housed in groups of three per cage at 22 ± 2 °C, provided with standard food and water, and maintained under pathogen-free conditions as per the animal care protocol. These experiments were approved by the Animal Welfare and Ethics Committee of the Biomedical Center at Qingdao University, and all procedures complied with the institutional regulations for the use and care of animals. Approximately 2 weeks later, infection was induced by the intramuscular injection of 10^7^ CFU bacterial suspension, PBS, or LPS into the right/left hind limb area of each SD rat (6 weeks old), establishing infection, blank control, and sterile inflammation control models, respectively.

### 2.7. In Vivo MRI

Different groups of animals were intravenously injected with Malt-MNPs or MNPs (2 mg/mL) via the tail vein, followed by MRI scanning using SIGNA Pioneer 3.0T (General Electric Company, Boston, MA, USA). The scanning parameters were as follows: TR = 3893 ms, slice thickness = 3 mm, TE = 119.6 ms, FA = 142, Acq = 2.63, BW = 244 Hz, and acquisition time = 96 s. The region of interest grayscale values were obtained using MRI software (U_VIEWER, Version R001.0.1.2, Shanghai United Imaging Healthcare Co., Ltd., Shanghai, China). SD rats were anesthetized and placed in the receiving coil. MRI scans were performed before injection and 3 h, 6 h, 12 h, and 24 h after the injection of MNPs or Malt-MNPs. After obtaining the MRI scans, muscle tissues from the infection site were collected, fixed in 4% PFA solution, paraffin-embedded, sectioned, and stained with Prussian blue.

### 2.8. Toxicity Assessment of Malt-MNPs

The cytotoxicity of Malt-MNPs was evaluated using the CCK8 assay kit. L929 cells were obtained from the laboratory. Cells in the logarithmic growth phase were digested and counted; consequently, they were seeded at a density of 5000 cells/well in a 96-well plate. After cells adhered to the plate, complete culture medium containing different concentrations of materials (MNPs or Malt-MNPs at 20, 40, 60, 80, or 100 μg/mL) was added. In total, 100 μL was added to each well, with 3 replicates per group. After 24 and 48 h, the CCK8 cytotoxicity assay was performed. After incubating with the CCK8 reagent for 2–4 h, the supernatant was collected and centrifuged into a new 96-well plate, and the absorbance at 450 nm was measured using a microplate reader. For hemolysis testing, 500 μL fresh rat arterial blood was collected in a heparinized centrifuge tube and diluted with 5 mL PBS. After centrifugation at 4000 rpm, the red blood cells (RBCs) were washed with physiological saline until the supernatant was clear. The supernatant was discarded, and the RBCs were resuspended in 10 mL PBS. In total, 200 μL RBC suspension was mixed with 800 μL PBS as the negative control group; 200 μL RBC suspension was mixed with 800 μL Triton X-100 as the positive control group; and 200 μL RBC suspension was mixed with 800 μL PBS containing 10–80 μg/mL Malt-MNPs as the experimental group. The samples were incubated at 37 °C for 1 h. After centrifugation, 100 μL supernatant was transferred to a 96-well plate, and the absorbance at 540 nm was measured using a microplate reader. Additionally, to assess the long-term in vivo biocompatibility of Malt-MNPs, the major organs (heart, liver, spleen, lungs, and kidneys) of rats were collected 10 days after the tail vein injection of PBS or Malt-MNPs. These organs were fixed in 4% PFA solution, paraffin-embedded, sectioned, and stained with H&E for histological analysis.

### 2.9. Statistical Analysis

Statistical analysis was performed using OriginPro 2024b software (OriginLab Corporation, Northampton, MA, USA). One-way analysis of variance (ANOVA) was used to determine statistical significance, with *p*-values of *p* < 0.05, *p* < 0.01, and *p* < 0.001 indicating significance (* representing *p* < 0.05, ** representing *p* < 0.01, *** representing *p* < 0.001, and **** representing *p* < 0.0001). The error bars represent the standard deviations obtained from three independent measurements.

## 3. Results

### 3.1. Characterization of Malt-MNPs

**Figure 1:**
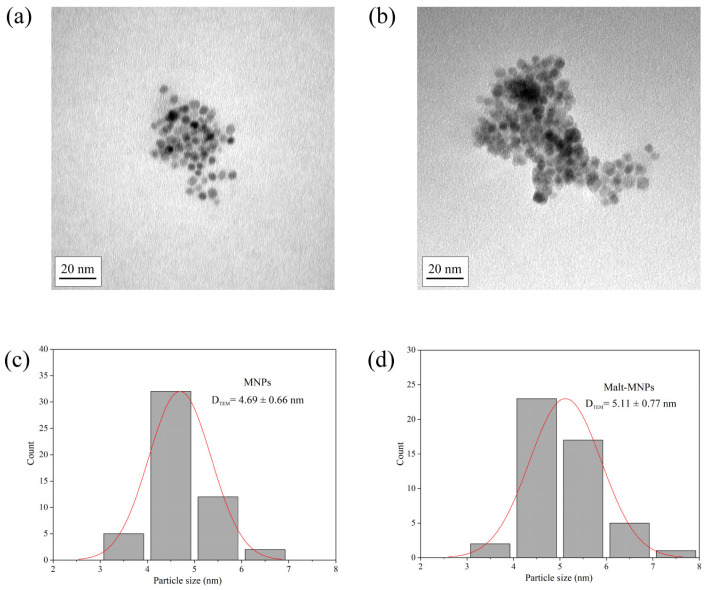
TEM images of MNPs (**a**) and Malt-MNPs (**b**). Particle size distribution and normal distribution curves (**c**,**d**).

We synthesized MNPs and conjugated them with maltotriose via a Schiff base reaction to produce Malt-MNPs. The transmission electron microscopy (TEM) images of MNPs (Figure 1a) and Malt-MNPs (Figure 1b) exhibited a spherical shape and uniform size distribution. The average diameter of MNPs, calculated using ImageJ 1.46r, was 4.69 ± 0.66 nm (Figure 1c), whereas that of Malt-MNPs slightly increased to 5.11 ± 0.77 nm (Figure 1d).

**Figure 2:**
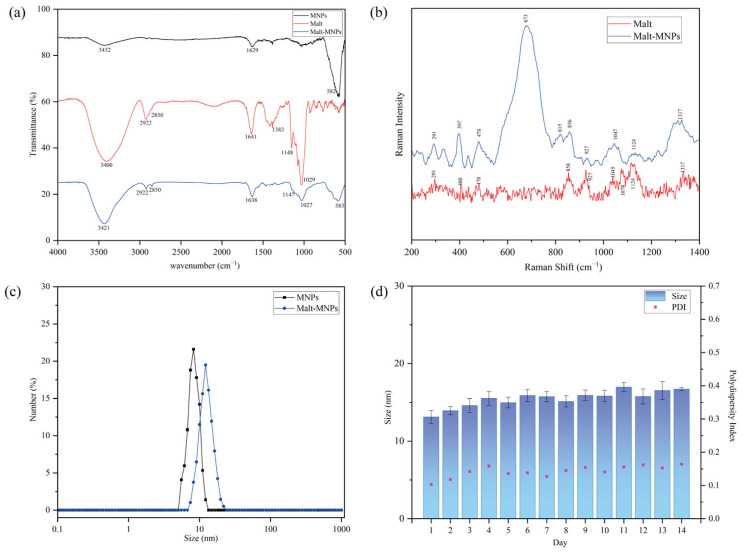
Characterization of Malt-MNPs. (**a**) FTIR spectra of MNPs (black), maltotriose (red), and Malt-MNPs (blue). (**b**) Raman spectra of maltotriose (red) and Malt-MNPs (blue). (**c**) DLS measurements of MNPs (black) and Malt-MNPs (blue). (**d**) Hydrodynamic size changes in Malt-MNPs measured over 14 consecutive days.

The grafting of maltotriose onto MNPs was confirmed using Fourier transform infrared (FTIR) spectroscopy (Figure 2a). The strong band at 582 cm^−1^ in bare MNPs corresponds to Fe–O stretching vibrations in the Fe_3_O_4_ lattice. The bands at 3432 cm^−1^, 1629 cm^−1^, and 1383 cm^−1^ are associated with adsorbed water or undissociated –OH groups on the surface of MNPs. In comparison, Malt-MNPs showed a similar Fe–O stretching vibration band at 583 cm^−1^. The broad band centered at 3421 cm^−1^ is attributed to N–H stretching vibrations from the reaction or –OH groups in maltotriose. The absorption bands at 2922 cm^−1^ and 2850 cm^−1^ correspond to C–H stretching vibrations, and the band near 1638 cm^−1^ arises from N–H deformation vibrations. The bands at 1147 cm^−1^ and 1027 cm^−1^, associated with C–N stretching vibrations, match the characteristic peaks of maltotriose, confirming the successful grafting of maltotriose onto MNPs. To verify the crystal phase composition of Malt-MNPs, the Raman spectroscopy results (Figure 2b) confirm that the core of Malt-MNPs is predominantly composed of magnetite (Fe_3_O_4_). The spectrum shows a strong peak at 673 cm^−1^, corresponding to the A1g mode of magnetite, which represents the symmetric stretching of Fe–O bonds. The sharpness of this peak suggests that the core is mainly magnetite rather than maghemite (γ-Fe_2_O_3_), which typically presents a shifted or broadened peak. Additional peaks at 291 cm⁻^1^ and 478 cm^−1^ characteristic of the T2g modes of magnetite further support this identification. Peaks at 815 cm^−1^, 856 cm^−1^, and 927 cm^−1^ indicate C–O–C stretching and glycosidic linkage vibrations in carbohydrates, confirming the presence of maltotriose on the nanoparticle surface. The similarity of these peaks in both the pure Malt and Malt-MNP spectra suggests that the coating is intact. Peaks at 1047 cm^−1^ and 1124 cm^−1^ correspond to C–O and C–C stretching in polysaccharides, associated with the glycosidic bonds in maltotriose, indicating that the coating retains its structure. The peak at 1317 cm^−1^, related to CH_2_ bending and CH deformation in carbohydrates, confirms that the organic coating is derived from maltotriose rather than other contaminants. Moreover, the average zeta potential of bare MNPs dispersed in ultrapure water was measured as +34.18 mV. After modification with maltotriose, the average zeta potential of Malt-MNPs slightly decreased to approximately +27.91 mV. Dynamic light scattering (DLS) measurements (Figure 2c) revealed that the hydrodynamic size of Malt-MNPs dissolved in ultrapure water was 12.16 nm/PDI = 0.103, slightly larger than that of MNPs (8.22 nm/PDI = 0.086). The apparent difference between the particle size distributions obtained from TEM and DLS is due to a fundamental difference in the measurement principles of the two techniques. TEM captures the physical diameter of an inorganic core without taking into account any surface coating or hydrodynamic effects. In contrast, DLS measures the hydrodynamic diameter of nanoparticles in suspension, which includes not only the core size but also the coating thickness, adsorbed solvent molecules, and the particle’s dynamic interaction with the surrounding medium. As a result, the hydrodynamic size is typically larger than that observed by TEM. Additionally, to assess the stability of Malt-MNPs, they were monitored over two weeks using DLS. The results (Figure 2d) showed that they remained stable with no notable aggregation observed over the 14-day period. The PDI values also showed a modest increase, ranging from 0.103 to 0.164, but remained below 0.2, which is generally considered to indicate good dispersion stability and a relatively uniform particle distribution.

### 3.2. Magnetic Properties and T2 of Malt-MNPs

**Figure 3:**
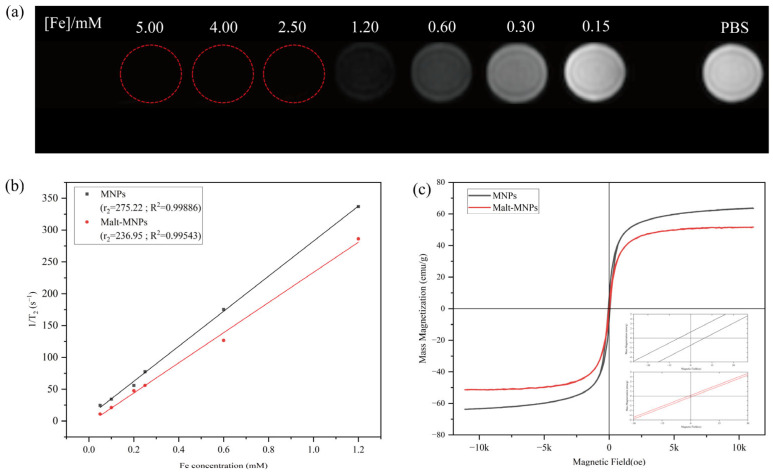
Magnetic properties and T2 of Malt-MNPs. (**a**) T2-weighted MRI images of Malt-MNPs at various Fe concentrations. (**b**) Transverse relaxivity (r2) efficiency of MNPs and Malt-MNPs. (**c**) Magnetic hysteresis curves of MNPs and Malt-MNPs. The insets show the low-field interval of the magnetization curve (black for MNPs, red for Malt-MNPs).

Figure 3a shows the T2-weighted MRI images of Malt-MNPs dispersed in PBS at various Fe concentrations. As the Fe concentration increases, the grayscale intensity in the MRI images decreases progressively, indicating that Malt-MNPs considerably influence T2. To further evaluate the transverse relaxivity (r2) of Malt-MNPs, T2 values were measured at different Malt-MNP concentrations (Figure 3b). The results demonstrated that the r2 of both MNPs and Malt-MNPs increased with the Fe ion concentration. The fitted r2 values were 275.22 mM^−1^·s^−1^ for MNPs and 236.95 mM^−1^·s^−1^ for Malt-MNPs. The measured coercive force (Hc) for MNPs was 9.66 Oe and for Malt-MNPs was 1.31 Oe. The remanent magnetization (Mr) was 1.27 emu/g for MNPs and 0.17 emu/g for Malt-MNPs. We conducted T2 relaxation time measurements on these samples again after 2 months of storage to assess their long-term stability. The newly measured r_2_ value was 227.94 ± 4.17 mM^−1^·s^−1^, which showed only a slight decrease compared to the original value of 236.95 mM^−1^·s^−1^. This minimal reduction indicates that Malt-MNPs exhibit excellent long-term stability. Figure 3c presents the magnetic hysteresis curves of MNPs and Malt-MNPs measured at room temperature. They both exhibit negligible coercivity and remanence, indicating their superparamagnetic properties. The saturation magnetization values (Ms) were 63.5 emu/g for MNPs and 51.6 emu/g for Malt-MNPs. The reported saturation magnetization values for Fe_3_O_4_ nanoparticles typically range from 60 emu/g to 80 emu/g. The decrease in Ms for Malt-MNPs is attributed to the nonmagnetic nature of maltotriose, which reduces the surface volumetric magnetic anisotropy and introduces rotational hindrance [53]. While the Ms decrease may affect the efficiency of the contrast generation in T2-weighted imaging, the targeted delivery to the infection site can compensate for this loss in part. Targeting efficiency is more critical in achieving effective imaging. Nevertheless, the magnetic hysteresis results confirm that both materials retain strong magnetic responsiveness and superparamagnetis [32].

### 3.3. In Vitro Uptake of Malt-MNPs by Bacteria

**Figure 4:**
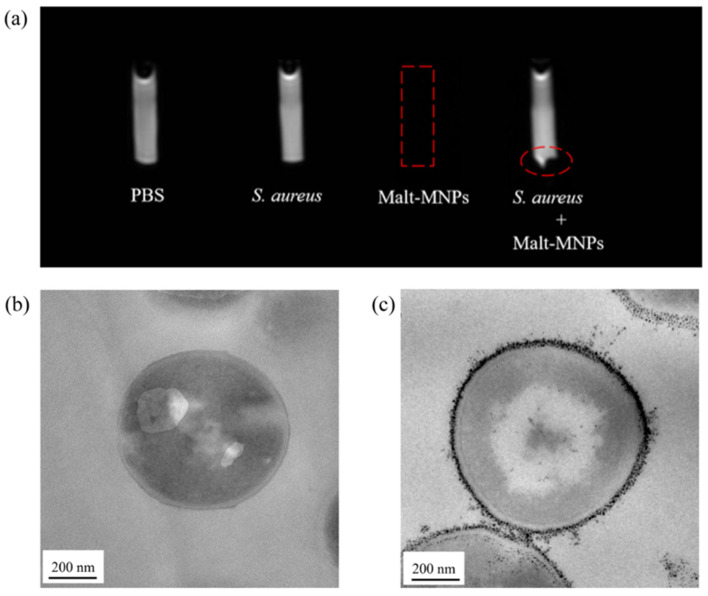
The in vitro uptake of Malt-MNPs by Staphylococcus aureus (*S. aureus*). (**a**) T2-weighted MRI images of PBS, *S. aureus* suspension, Malt-MNP solution, and the solution obtained after the co-incubation of *S. aureus* and Malt-MNPs; (**b**) A thin-sectioned TEM image of *S. aureus*; (**c**) a thin-sectioned TEM image obtained following the co-incubation of *S. aureus* and Malt-MNPs.

We conducted MRI and ultrathin-sectioned TEM analyses on mixed solutions after co-incubating Malt-MNPs with *S. aureus* suspensions for 1 h to evaluate the imaging capability of Malt-MNPs. T2-weighted MRI images (Figure 4a) were obtained for PBS, *S. aureus* suspension (10^7^ CFU), Malt-MNP solution (2.5 mM), and the mixed solution of *S. aureus* and Malt-MNPs. The MRI signal intensities (SIs) of PBS and the bacterial suspension showed no notable difference. However, the MRI image of the Malt-MNP solution (indicated by the rectangular dashed line) exhibited an extremely low SI, indicating the strong relaxation capability of Malt-MNPs and confirming their excellent dispersion. Interestingly, for Malt-MNPs co-incubated with *S. aureus*, the upper portion of the centrifugal tube displayed an SI similar to those of PBS and the *S. aureus* suspension. In contrast, the bottom portion of the EP tube showed a precipitate with an extremely low SI (indicated by the elliptical dashed line). This phenomenon is due to the robust binding and internalization of Malt-MNPs by *S. aureus*, which causes the bacterial cells to sediment at the bottom, forming a low-signal precipitate. To further validate these findings, TEM analyses were performed on bacterial pellets obtained by centrifuging the *S. aureus* suspension and the co-incubated mixture. The TEM image of pure *S. aureus* (Figure 4b) revealed smooth, round bacterial cells. In contrast, the TEM image of *S. aureus* co-incubated with Malt-MNPs (Figure 4c) showed substantial Malt-MNP adhesion on the bacterial cells, in addition to minor internalization within the cells, indicating that *S. aureus* can effectively capture Malt-MNPs in solution and gradually internalize them during the co-incubation process. These results confirm the bacterial targeting capability of Malt-MNPs and demonstrate their potential for MRI-based bacterial identification.

### 3.4. In Vivo Imaging Capability of Malt-MNPs at Infection Sites

**Figure 5:**
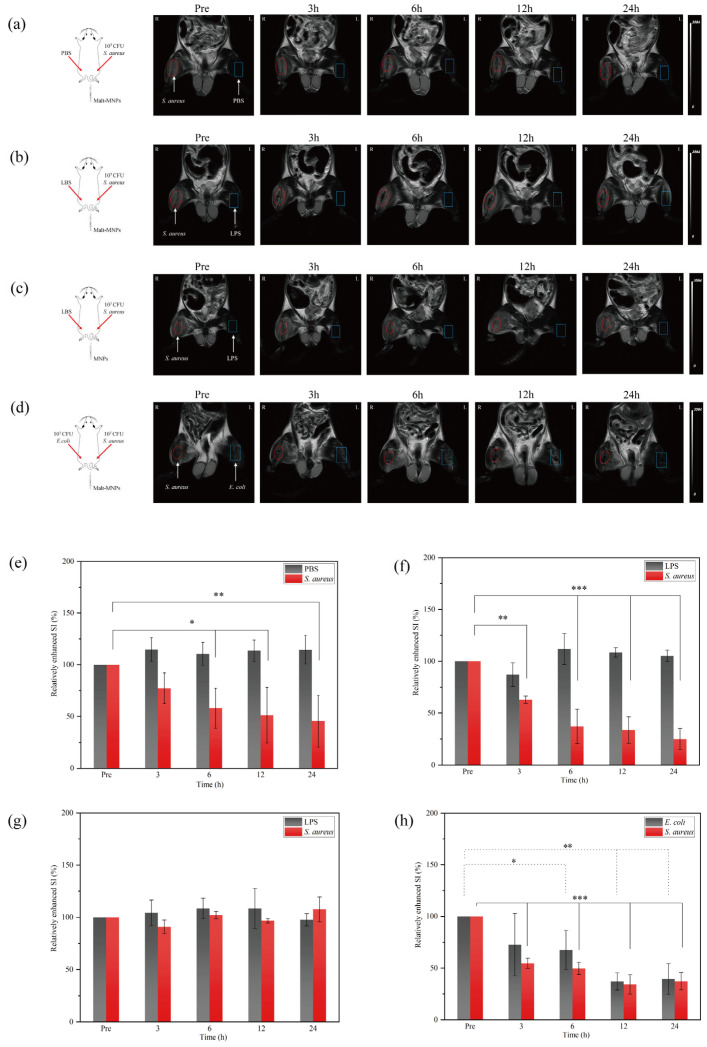
In vivo MRI. (**a**) The in vivo MRI of *S. aureus* infection sites (right red areas) and PBS control (left blue areas) in rats before and 3, 6, 12, and 24 h after the tail vein injection of Malt-MNPs. (**b**) The in vivo MRI of *S. aureus* infection sites (right red areas) and LPS-induced sterile inflammation control (left blue areas) in rats before and 3, 6, 12, and 24 h after the tail vein injection of Malt-MNPs. (**c**) The in vivo MRI of *S. aureus* infection sites (right red areas) and LPS-induced sterile inflammation control (left blue areas) in rats before and 3, 6, 12, and 24 h after the tail vein injection of MNPs. (**d**) The in vivo MRI of *S. aureus* infection sites (right red areas) and *E. coli* infection sites (left blue areas) in rats before and 3, 6, 12, and 24 h after the tail vein injection of Malt-MNPs. (**e**–**h**) Relative signal intensity enhancement in the regions of interest of the T2-weighted MRI results corresponding to the four groups in (**a**–**d**) showing statistically significant differences (* denotes *p* < 0.05, ** denotes *p* < 0.01, and *** denotes *p* < 0.001).

We established a corresponding bacterial infection model in Sprague Dawley (SD) rats to demonstrate the potential of Malt-MNPs for in vivo bacterial imaging. We injected 500 μL of *S. aureus* (1 × 10^7^ CFU) and 500 μL of PBS into the right and left lower limb muscles of the mice, respectively, to create a stable right lower limb infection model and a contralateral control (Figure 5a). In Figure 5b, 500 μL of *S. aureus* (1 × 10^7^ CFU) and 500 μL of Lipopolysaccharide (LPS) (1 mg/mL) were injected into the right and left lower limb muscles of the mice, respectively, to construct a stable right lower limb *S. aureus* infection site and a left lower limb sterile inflammation region. For both groups, 1 mL of Malt-MNPs (2 mg/mL) was injected via the tail vein before MRI scanning. As shown in Figure 5c, the same model was used for the injection of 1 mL of MNPs (2 mg/mL) for comparison. To further investigate the broad-spectrum uptake of Malt-MNPs by Gram-positive and Gram-negative bacteria, the injection of 500 μL of *S. aureus* (1 × 10^7^ CFU) and 500 μL of *E. coli* (1 × 10^7^ CFU) into the right and left lower limb muscles of the mice was employed, respectively, to create stable right and left lower limb infections with *S. aureus* and *E. coli* (Figure 5d). Before MRI scanning, 1 mL of Malt-MNPs (2 mg/mL) was injected via the tail vein.

The T2-weighted MRI results revealed no notable SI differences at the right lower limb *S. aureus* infection site between pre-injection and 3 h after injection (Figure 5a). However, 6–24 h after injection, the SI at the infection site notably decreased compared to the pre-injection SI, whereas the left lower limb PBS control group showed no prominent changes during the 24 h period. Figure 5b supports these results, where the *S. aureus* infection site showed a noticeable decrease in SI within 3 h of injection. In Figure 5c, no notable imaging changes were observed on either side because MNPs (without maltotriose coating) failed to accumulate stably at the infection sites, being cleared quickly through the bloodstream. As shown in Figure 5d, the bilateral lower limbs included both Gram-positive and Gram-negative bacteria. Both lower limbs showed remarkable SI changes after 3 h of injection, demonstrating the broad-spectrum diagnostic potential of Malt-MNPs.

We further analyzed the regions of interest of the T2-weighted MRI results of the bilateral lower limbs of rats from each group, obtaining the SI and evaluating the relative enhanced SI using the following formula, Relative Enhanced SI = (SI_t_/SI_pre_) × 100%, where SI_pre_ and SI_t_ represent the SIs before and after t hours of probe injection, respectively (Figure 5e–h). In Figure 5e, the relative enhanced SI of the right lower limb *S. aureus* infection site decreased from 100 (before injection) to 77.25 ± 14.89 (*p* = 0.18) at 3 h, 57.96 ± 19.32 (*p* = 0.026) at 6 h, 51.21 ± 26.73 (*p* = 0.012) at 12 h, and 45.40 ± 24.91 (*p* = 0.007) at 24 h. The left lower limb PBS control group showed no significant differences over time, with relative enhanced SI values increasing from 100 (before injection) to 114.76 ± 11.48 (*p* = 0.11) at 3 h, 110.48 ± 10.92 (*p* = 0.25) at 6 h, 113.58 ± 10.28 (*p* = 0.14) at 12 h, and 114.53 ± 13.80 (*p* = 0.12) at 24 h. As shown in Figure 5f, the relative enhanced SI of the right lower limb *S. aureus* infection site decreased from 100 (before injection) to 62.92 ± 3.50 (*p* = 0.0015) at 3 h, 37.24 ± 16.66 (*p* < 0.001) at 6 h, 33.70 ± 12.71 (*p* < 0.001) at 12 h, and 25 ± 10.09 (*p* < 0.001) at 24 h. The left lower limb LPS sterile inflammation group showed no significant differences, with the relative enhanced SI values changing from 100 (before injection) to 87.07 ± 11.34, 111.84 ± 14.92, 108.48 ± 4.54, and 125.16 ± 34.25 (all *p* > 0.05) at 3, 6, 12, and 24 h of injection, respectively, indicating significant differences in the relative enhanced SI for the *S. aureus* infection site, while no significant differences were observed at the sterile inflammation site. The relative enhanced SI differences before and after the tail vein injection of MNPs showed no statistical significance (Figure 5g). In Figure 5h, the relative enhanced SI of the right lower limb *S. aureus* infection site decreased from 100 (before injection) to 54.68 ± 4.86 (*p* < 0.001), 49.63 ± 5.93 (*p* < 0.001), 34.22 ± 9.37 (*p* < 0.001), and 37.35 ± 8.25 (*p* < 0.001) at 3 h, 6 h, 12 h, and 24 h of injection, respectively, whereas the left lower limb *E. coli* infection site showed a gradual decrease from 100 (before injection) to 76.68 ± 30.29 (*p* = 0.09), 67.68 ± 18.76 (*p* = 0.049), 37.15 ± 8.22 (*p* = 0.001), and 39.56 ± 14.83 (*p* = 0.002) at 3 h, 6 h, 12 h, and 24 h of injection, respectively, demonstrating significant differences in SI changes at both *S. aureus* and *E. coli* infection sites after Malt-MNP injection.

**Figure 6:**
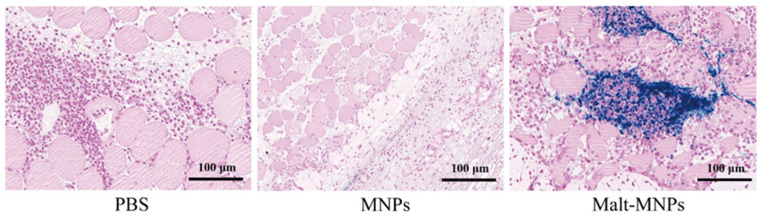
Representative Prussian blue staining images of infection foci after 24 h of injection for the blank, MNP, and Malt-MNP groups.

Additionally, the pathological examination of the *S. aureus* infection sites after 24 h of PBS, MNP, and Malt-MNP injection (Figure 6) showed iron accumulation at the infection sites treated with Malt-MNPs following Prussian blue staining. In contrast, no notable iron accumulation was observed in the PBS and MNP groups. These results confirm that Malt-MNPs can accurately target bacterial infection sites and markedly differentiate bacterial infection sites from sterile inflammation sites. Moreover, the introduction of maltotriose enhances the stability and long-lasting accumulation of MNPs at infection sites, facilitating further investigation or treatment.

### 3.5. Toxicity Evaluation of Malt-MNPs

**Figure 7:**
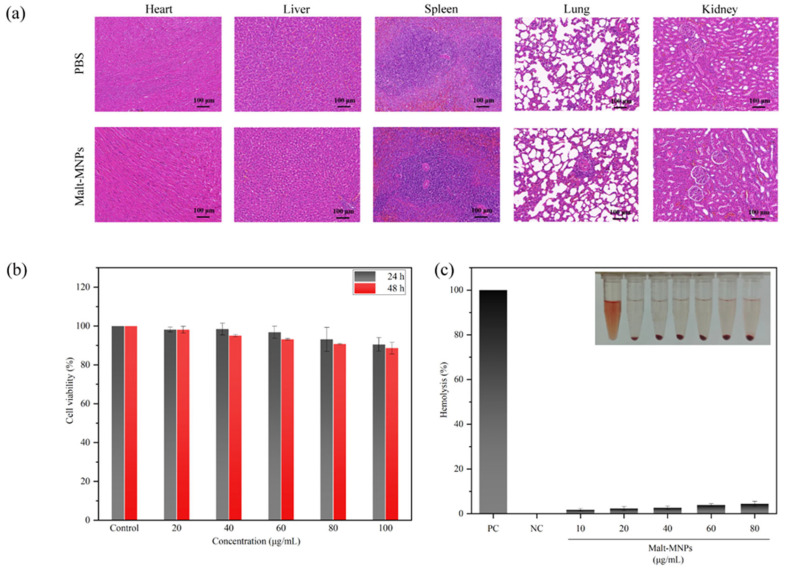
In vivo and in vitro toxicity testing of Malt-MNPs. (**a**) Histological evaluation of tissues from various organs (heart, liver, spleen, lung, and kidneys) of healthy rats treated with the tail vein injection of PBS or Malt-MNPs for 10 days. Scale bar: 100 μm. (**b**) Cell viability of L929 cells treated with Malt-MNPs at various concentrations for 24 h and 48 h. (**c**) Hemolysis rate and images (inset) after the incubation of rat blood erythrocyte suspension with Malt-MNPs at various concentrations for 1 h. The positive control (PC) was treated with Triton X-100, and the negative control (NC) was treated with PBS.

After 10 days of treatment, the rats injected with PBS served as the blank control. Histological sections of the heart, liver, spleen, lung, and kidneys were stained with hematoxylin and eosin (HE) and analyzed, as shown in Figure 7a. The HE-stained tissue sections from both groups exhibited normal tissue morphology with no notable differences, indicating that the in vivo toxicity of Malt-MNPs is negligible. The cytotoxicity of Malt-MNPs was evaluated using the established Cell Counting Kit-8 (CCK-8) assay. As shown in Figure 7b, when L929 cells were incubated with different concentrations of Malt-MNPs for 24 and 48 h, the cell viability did not greatly decrease. Even at the high concentration of 100 μg/mL, the cell viability remained above 90%, suggesting that Malt-MNPs exhibit low cytotoxicity toward rat cells, supporting their potential for clinical applications. Figure 7c shows no remarkable hemolysis in vitro for Malt-MNPs. Even at high concentrations, the hemolysis rate was only 4.43%, indicating that Malt-MNPs possess good blood compatibility.

## 4. Discussion

In this study, we developed maltotriose-functionalized magnetic nanoparticles (Malt-MNPs) and investigated their properties as well as their diagnostic value in the early detection of bacterial infections using MRI. Generally speaking, to achieve targeted diagnosis of lesions, imaging agents should meet three key criteria: (1) possess highly specific targeting groups; (2) contain functional groups that can generate contrast differences; and (3) exhibit high biocompatibility. However, naked MNPs rely on passive distribution and are quickly cleared from the bloodstream, limiting their retention at infection sites. In contrast, Malt-MNPs actively target bacterial infection sites via the maltodextrin transporter system, achieving localized retention and sustained T2 signal reduction. The r_2_ relaxivity of iron oxide nanoparticles is primarily determined by their ability to generate local magnetic field inhomogeneities, which accelerate the dephasing of surrounding water protons. In our study, the unmodified MNPs exhibited an r_2_ relaxivity of 275.22 mM^−1^·s^−1^, whereas the Malt-MNPs showed an r_2_ value of 236.95 mM^−1^·s^−1^. These values are favorable when compared to previously reported data. For instance, Tan et al. reported an r_2_ value of 228 s^−1^·mM^−1^ Fe [54], Weis et al. observed an r_2_ value of 236 s^−1^·mM^−1^ Fe [55], Fang et al. measured an r_2_ value of 339 s^−1^·mM^−1^ Fe [56], and Borase et al. reported a considerably lower r_2_ of 62 s^−1^·mM^−1^ Fe [57]. Additionally, clinically used superparamagnetic iron oxide nanoparticles (SPIONs) such as Feridex (r_2_ = 98.3 mM^−1^·s^−1^) and Ferumoxtran-10 (r_2_ = 60 mM^−1^·s^−1^) exhibit significantly lower relaxivity, with that of ferucarbotran (239 ± 2 s^−1^·mM^−1^ Fe) being only slightly higher than that of our Malt-MNPs [58,59]. The maltotriose coating improves colloidal stability and reduces aggregation under physiological conditions, as demonstrated by the highly stable hydrodynamic size over 14 days. This stability is critical for ensuring reproducible imaging performance.

The slight decrease in the r_2_ value over time is likely due to minor aggregation effects, which are difficult to completely avoid in colloidal nanoparticle systems. Particle aggregation can induce dipole–dipole interactions, potentially leading to changes in the superparamagnetic blocking volume and affecting the relaxation properties. However, our maltotriose coating strategy appears to effectively limit significant dipole–dipole interactions between iron cores as the coating layer reduces direct core–core collisions. To further understand this behavior, we compared our findings to previous studies on the long-term stability of similar iron oxide-based nanoparticles. For instance, Masoudi et al. [60]. observed that nanoparticles with amorphous or insufficient oxide shells experienced the substantial oxidation of the metallic iron cores over several months, leading to significant reductions in magnetic properties and MRI relaxivity. Conversely, nanoparticles with thicker and crystalline oxide shells exhibited markedly improved stability and retained consistent relaxivity over prolonged periods. In our study, the maltotriose-functionalized nanoparticles effectively maintained their structure and prevented significant oxidation, as evidenced by the consistent hydrodynamic diameters measured by DLS over the storage period. Therefore, we infer that the small decrease in r_2_ is not primarily driven by oxidative deterioration but rather by mild and limited nanoparticle aggregation. Additionally, the maltotriose coating introduces a hydrophilic layer that increases the effective hydrodynamic diameter and provides steric hindrance around the magnetic core. This restricted access reduces the efficiency of water proton interactions with the core surface, leading to a modest decrease in the r_2_ relaxivity of Malt-MNPs compared to that of bare MNPs. Similar observations have been reported by Weis et al. and Borase et al., where hydrophilic coatings were found to increase the hydrodynamic diameter and influence the relaxation behavior of magnetic nanoparticles by limiting water proton interactions with the core [55,57,59]. Furthermore, Wang et al. demonstrated that particle size plays a critical role in determining r_2_ relaxivity, highlighting the need to strike a delicate balance between maximizing magnetic effects and maintaining favorable biodistribution properties for biomedical applications.

Our results indicate that Malt-MNPs exhibit specific accumulation at bacterial infection sites, allowing for accurate MRI signal enhancement within 3–6 h of injection. The selective accumulation of Malt-MNPs at bacterial infection sites can be attributed to two factors: the specific uptake of maltotriose by bacteria and the increased vascular permeability and immune cell recruitment in the infectious microenvironment [61]. Interestingly, compared to the group without sterile inflammation on the contralateral side (Figure 5e), the infection group with sterile inflammation on the contralateral side (Figure 5f) demonstrated faster nanoparticle accumulation dynamics at the infection site. This could be due to the exacerbation of the overall inflammatory burden in the animal model, where contralateral sterile inflammation indirectly enhanced microcirculatory permeability at the infection site through systemic inflammatory mediators, facilitating nanoparticle penetration. This was further confirmed by changes in MRI SI in the bilateral bacterial infection model (Figure 5g), which showed faster signal changes compared to unilateral infection. Biocompatibility studies (Figure 7) revealed that the cell toxicity, hemolysis, and organ toxicity of Malt-MNPs were negligible. The histological analyses showed no prominent tissue damage, and high cell viability (above 90% cell viability) at high nanoparticle concentrations emphasized their safety for clinical applications. These findings agree with previous studies demonstrating the low toxicity of iron oxide-based nanoparticles [62].

Imaging modalities such as fluorescence imaging, photoacoustic imaging, PET, and SPECT have been recently investigated for bacterial infection imaging using corresponding contrast agents as they provide methods complementary to traditional imaging techniques [20,63,64,65]. However, these methods have limitations, such as poor penetration depth, photobleaching, nonspecific background signals, or radiation. In this study, MRI was selected owing to its unique advantages, including high resolution, excellent imaging depth, and noninvasiveness [39]. Regardless, the animal model used in this study may not fully replicate the complexity of bacterial infections in human patients. Future studies will validate the diagnostic performance of Malt-MNPs in larger, more clinically relevant models. Moreover, although the bacterial uptake of maltotriose was confirmed, the potential off-target effects of Malt-MNPs in other tissues need further investigation. Future research will integrate Malt-MNPs with other diagnostic and therapeutic strategies to enhance their clinical utility. For example, MNPs can serve as carriers for therapeutic agents, enabling concurrent diagnosis and therapy. Moreover, optimizing the surface modification of Malt-MNPs to enhance their biocompatibility and reduce potential immunogenicity will help translate them into clinical practice, a key focus of our future work.

## 5. Conclusions

In this study, we successfully synthesized maltotriose-functionalized magnetite nanoparticles (Malt-MNPs) and evaluated their colloidal stability, magnetic properties, bacterial-specific targeting, and biocompatibility. The DLS and TEM results demonstrated that Malt-MNPs possess a small particle size and exhibit good dispersion. Small-sized magnetite nanoparticles are prone to oxidation into maghemite in environmental conditions, leading to a decrease in saturation magnetization (Ms). To confirm the crystalline phase composition of Malt-MNPs, we performed Raman spectroscopy, which revealed that the core of the nanoparticles remains to be predominantly magnetite without significant oxidation. This finding suggests that the relatively low Ms values observed in our experiments are more likely due to size-dependent effects rather than a significant phase transition from magnetite to maghemite. The magnetic characterization results indicated that bare MNPs exhibit slightly higher coercivity and remanent magnetization, which could be attributed to dipole–dipole interactions between the nanoparticles, thereby increasing the effective superparamagnetic blocking volume through enhanced magnetic anisotropy. In contrast, Malt-MNPs showed excellent superparamagnetic properties with nearly no coercivity and remanent magnetization, likely due to the maltotriose coating, which effectively shields the dipole–dipole interactions between the MNPs. More importantly, compared with conventional MNPs, the functionalization with maltotriose enhanced the ability of the nanoparticles to target bacterial infection sites through the maltodextrin transport system, which is uniquely expressed by bacteria. This specificity allowed Malt-MNPs to effectively distinguish bacterial infections from sterile inflammation regions, overcoming the key limitation of traditional MRI contrast agents that rely solely on passive accumulation in areas with increased vascular permeability. Further, the in vivo experiments provided evidence that Malt-MNPs can remain at bacterial infection sites for a prolonged period while rapidly clearing from sterile inflammation areas, thereby minimizing off-target effects and improving diagnostic accuracy. The next focus of our work needs to be on the long-lasting retention of Malt-MNPs in bacteria using more advanced in vitro models, such as 2D/3D microfluidic cell culture microarrays that can mimic the in vivo environment. In summary, our results suggest that Malt-MNPs hold great potential for clinical translation, offering a novel approach for the early, accurate, and noninvasive diagnosis of bacterial infections.

## Data Availability

The original contributions presented in this study are included in the article material. Further inquiries can be directed to the corresponding authors.
